# Sand Dune Encroachment and Desertification Processes of the Rigboland Sand Sea, Central Iran

**DOI:** 10.1038/s41598-017-01796-z

**Published:** 2017-05-08

**Authors:** Hesam Ahmady-Birgani, Kenneth G. McQueen, Mazaher Moeinaddini, Hamidreza Naseri

**Affiliations:** 1 Faculty of Natural Resources, Urmia University, Urmia, Iran; 2 IAE, Faculty of Education, Science, Technology and Mathematics, University of Canberra, Canberra, Australia; 3 Faculty of Natural Resources, University of Tehran, Karaj, Iran; 4 International Desert Research Center, University of Tehran, Tehran, Iran

## Abstract

Early studies on sand dune movement and desertification in Iran have not always been convincingly demonstrated because of problems with the field-based measurements. In some areas where various land uses have been engulfed by aeolian sand dunes, desertification is clear, but in other less settled areas, it may not be so obvious. The objective of this study is to demonstrate encroachments of the Rigboland sand sea, central Iran, in its different directions and variable magnitude rates. Determining the rate and direction of the sand sea movements is critical for specifying which lands should be prioritized and quickly protected. The study has trialed a change detection technique which uses a Cross-Tabulation module to compare two available Landsat_TM_ images over the Rigboland sand sea. This indicates that within a ten-year span (from 1988 to 1998) more than 200 ha/yr were added to the Rigboland sand sea, from the alluvial fan landforms in the eastern upstream, outer margins of the Rigboland sand sea. Coupled with GIS techniques, this type of analysis of the remote sensing (RS) images provides an effective tool for the monitoring and prognostication of sand dune movement and sand sea change.

## Introduction

Desertification is a type of land degradation in arid, semi-arid and dry sub-humid areas resulting from various factors, including climatic variation and human activity^1^. Sand movement by wind following desertification is a complex process involving several types of grain movement that occur more or less simultaneously^2^. Dune growth and movement is the result of sand flow on and around a dune during periods when the wind is strong enough to move sand. Aeolian dunes are constantly changing shape in response to changes in wind velocity or direction and they grow when more sand drifts onto them from surrounding areas than is removed downwind. Sand seas (Erg) are one of the most significant features created by wind deposition^3^ and are broad, flat areas of desert covered with wind-swept sand with little or no vegetative cover. Sand dunes within sand seas can change in distribution, extent, size (length or height), or form depending on wind direction and strength^4^. The formation, activity and stability of sand seas are closely related to climatic conditions and dunes may be reactivated or stabilized as conditions change^5^.

The ability to observe dune field patterns by remote sensing (RS) techniques has stimulated a shift away from the single-dune studies popular in the 1980s and 1990s^6^ towards dune field-scale studies that incorporate spatial analysis (SA) of boundary conditions, dune activity, dune patterns and hierarchies, and dune-dune relationships^7, 8^.

Improvements in the spectral and spatial resolution of RS data have substantially enhanced the ability of researchers to resolve dune features and processes, not so apparent in earlier types of imagery^9^. Improved analysis of RS data provides a suite of techniques for monitoring dust event occurrences^10^ and changes in sand dunes, especially in difficult to access sand seas, not easily achievable by field studies at a regular and wide coverage. Previous studies of other sites have documented sand sea encroachment and sand dune movements with the aid of RS and GIS data plus field-based surveying methods^9, 11–17^. These previous studies also indicate that the rate of sand sea development differs according to wind speed, wind direction, morphology of sand dunes, sand availability, as well as the location of the sand sea in terms of topographic position and geographical latitude.

**Figure 1:**
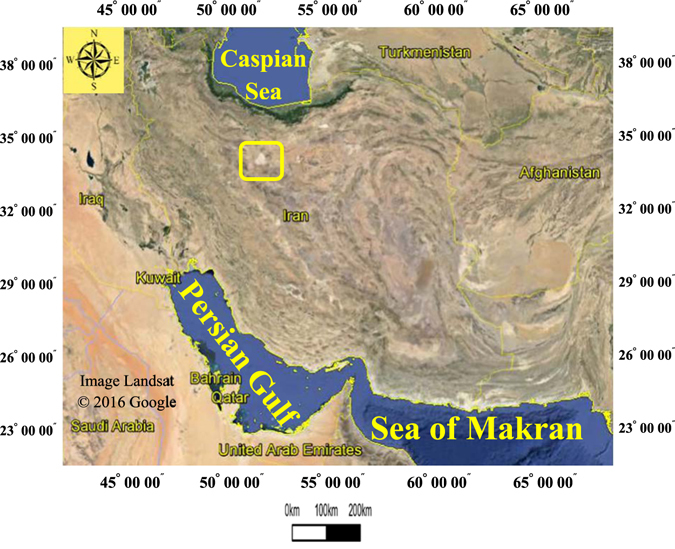
The location of the Rigboland sand sea shown by yellow rectangle in centre of Iran (Image from Google Earth Software).

The Rigboland sand sea is one of the largest sand seas in Iran (covering 1120 km^2^). It is located in the centre of Iran, 35 km east of Kashan city, between latitudes 33°50′38″ and 34°20′.14″ N and longitudes 51°30′55″ and 51°54′.80″ E. The sand sea is at an altitude of ca. 900 m above mean sea level (AMSL). It has a hot, dry climate with mean annual precipitation of around 100 mm, and mean annual temperature of 19 °C (Fig. 1).

**Figure 2:**
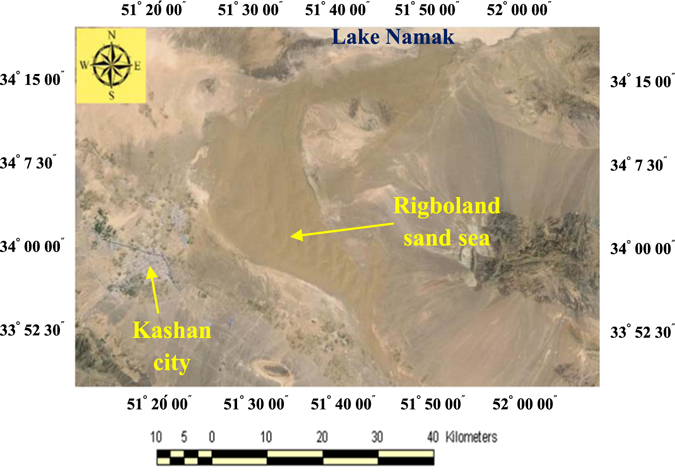
The confines of Rigboland sand sea with brown color in centre of Iran (ENVI software version 5.1).

The Rigboland sand sea contains a wide range of desert and arid landforms and aeolian features, including alluvial fans, sand dunes with different shapes and sizes, various desert bedform features, as well as different geomorphological facies related to playas (e.g. around Lake Namak or Salt Lake). The area also contains various land uses (e.g. residential areas, industrial parks, agricultural and orchard lands, rangelands) (Fig. 2).

The study reported here examines changes in area and shape of the Rigboland sand sea in Kashan, central Iran over a 10 year period. Improved general knowledge of the changing direction and magnitude of movement of the Rigboland sand sea will assist better decision making for urbanization and prioritization for control of growth and development.

## Results

### Image processing

**Figure 3:**
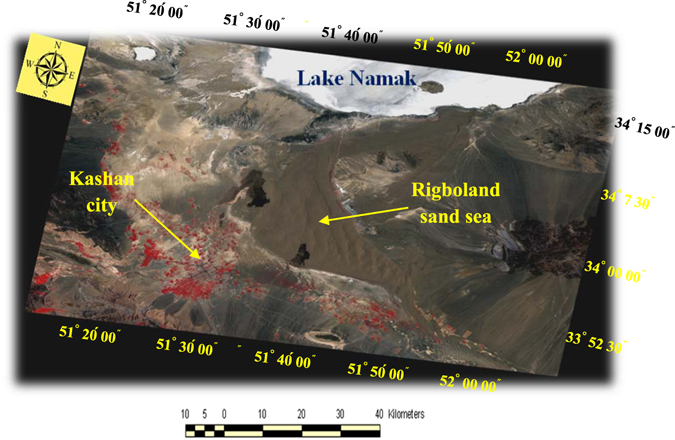
The Landsat_TM_ image of the Rigboland sand sea and surrounding areas with geometry and atmospheric corrections plus radiometric calibration (ENVI software version 5.1).

An example of a Landsat_TM_ satellite image after pre-processing, including geometry and atmospheric corrections, as well as radiometric calibrations, is shown in Fig. 3. It should be noted that for geometry correction a result of RMSE less than 0.5 pixel (~0.3 pixel) was achieved. In this study, four spectral bands were identified to make false color composites, including band numbers 1, 2, 5 and 7, leading to considerably higher overall accuracy than currently available in terms of spectral recognition of geomorphological features and land uses.

**Figure 4:**
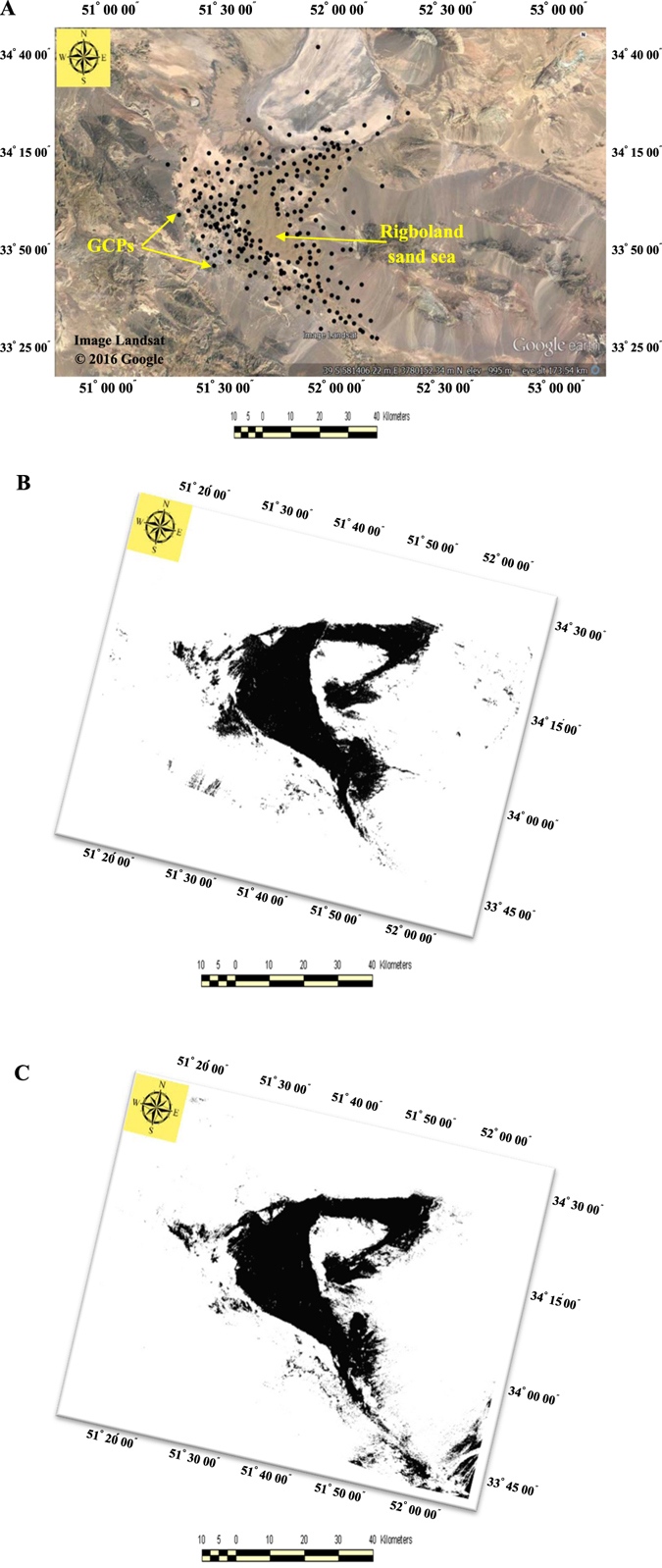
(**A**) Location (shown as black circles) of 250 ground control points (GCPs) for accuracy assessment of classification of the geomorphological features and land uses in the Rigboland sand sea. (**B**) 1988 Landsat_TM_ image classification through maximum likelihood classifier and (**C**) 1998 Landsat_TM_ image classification through maximum likelihood classifier. Comparison of the black shaded areas show changes of the Rigboland sand sea over the 10 years via image classification (IDRISI software version Kilimanjaro).

Following this assessment, representative areas of interest (AOI_s_) were selected as training sites for classification of the geomorphological features and land uses in the Rigboland sand sea. In applying the maximum likelihood classifier and for improving precision and accuracy of the results, two classes were defined comprising the Rigboland sand sea (the black shaded areas) and non-sand sea (the white-coloured areas) (Fig. 4B,C). Accuracy assessment was done with information from 250 ground control points (GCPs) to verify the results. The distribution and locations of the GCPs were selected for different land unit sites where there were significant changes in lithology, geomorphic features, land use and vegetation cover. They included areas where sand dune encroachment and desertification were obvious, sites of former villages, former agricultural lands now covered by thick sand and desert dunes (Fig. 4A).

### Rigboland Sand Sea encroachment determined by change detection technique

**Figure 5:**
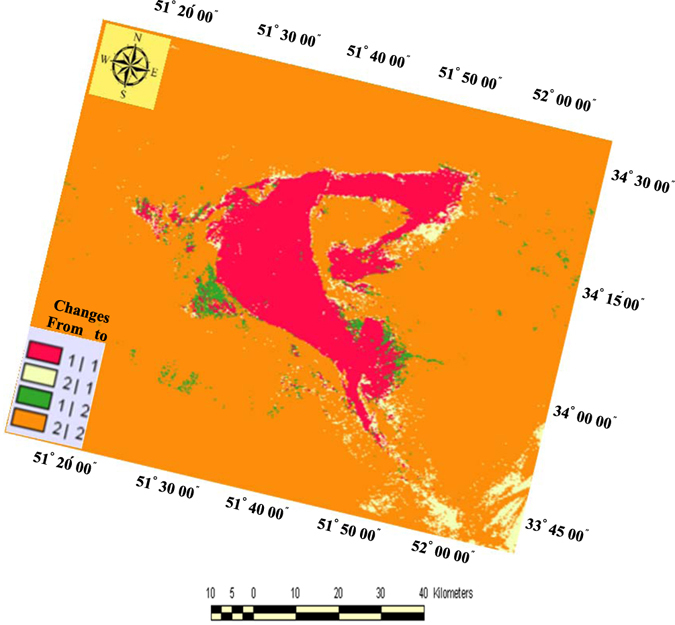
Changes detected between two Landsat_TM_ images using the cross-tabulation module. Red represents the Rigboland sand sea area without change. The yellow and green show areas added to and diminished from the Rigboland sand sea from other geomorphological features and land uses (i.e. residential areas, industrial parks, agricultural and orchard lands, rangelands) respectively over the period from 1988 to 1998 (Arc GIS software version 10.2).

To measure and monitor the direction and magnitude of changes in the Rigboland sand sea, a GIS-based method for extraction of sand sea encroachment was used. The results are summarized in Fig. 5 and Table 1. In Fig. 5, the areas of the Rigboland sand sea without change are shown in red, yellow shows areas that have been added to the sand sea from other geomorphological features and land uses (i.e. residential areas, industrial parks, agricultural and orchard lands, rangelands). The green areas are former parts of the Rigboland sand sea that have changed to other forms and land uses. The orange areas represent all geomorphological features and land uses apart from the Rigboland sand sea.

**Table 1:** The estimated rate of change detection technique for Rigboland sand sea.

The Type of Class	Change trend (from … to …)	Landsat_TM_ images pixel area (m^2^)	Class area (ha)
Sand sea	From 1 to 1	900	11632
Added to sand sea	From 2 to 1	900	3608
Reduced from sand sea	From 1 to 2	900	1492
Other landforms	From 2 to 2	900	86300

Given the number of pixels of each class (pixel displacement) and based on a 30-meter spatial resolution of Landsat_TM_ images, the area of all classes was calculated. The change trend of pixels in each class is shown in Table 1. The estimated change detection rate for the Rigboland sand sea over the 10 years from 1988 to 1999 is an addition of 3608 ha and reduction of 1492 ha with an average annual change rate of 212 ha/yr (Table 1). These results indicate that the Rigboland sand sea is mostly increasing in the southeastern and northeastern parts of the study area. The northwestern and western parts of the Rigboland sand sea are not currently sand dunes or sandy lands and the sand sea has been replaced by other geomorphological features and land uses (i.e. residential areas, industrial parks, agricultural and orchard lands) in these areas (Fig. 5).

The classification results need to be compared with the ground-truthing data in order to assess their accuracy. A classification error matrix was used for computing the overall Kappa coefficient. As Table 2 shows, the overall Kappa coefficient and overall accuracy values are 0.7917 and 87.46% respectively. Both overall Kappa coefficient and overall accuracy values of the results reflect the overall classification situation, which can provide the reliability of the classes of interest.

**Table 2:** The accuracy analysis of change detection technique for Rigboland sand sea.

Classes	The number of pixels		
	Sand sea	Others	Total
Sand sea	1292427	400919	1693346
Others	165791	9588887	9754678
Total	1458218	9989806	11448024
Overall Kappa coefficient	0.7917		
Overall accuracy (%)	87.46		

## Discussion

The purpose of this research was to test the application of a remote sensing method for determining the encroachment of the Rigboland sand sea. In the Rigboland sand sea, most aeolian landforms, including crescentic, linear and star dunes, plus nebkhas, zibars and sand sheets are present. Around the margins of the sand sea, longitudinal dunes are dominant, with some barchan dunes. These dunes are commonly found in wide unimodal or bi-directional wind regimes and occasionally occur in complex wind regimes^18, 19^. These two forms of sand dune (crescentic and linear) are grouped as very active sand dunes and move at highly varying rates and in various directions. Therefore, we believe, that when working on sand seas with their vast areas and fast changes in aeolian landforms and geomorphological features, there are major advantages in using RS coupled with GIS techniques. However, investigations that combine RS-based image analysis with field-based measurements are probably the most suitable and transferable to models, because they consider a range of spatial-temporal scales^9^.

Although the magnitude and direction of the Rigboland sand sea movement were measured using the RS technique in this study, previously Ahmady-Birgani^20^ used field-based surveying methods for measuring sand dune movement (i.e. sticks and pins). He was able to track the motion of the sand dunes and measured an average migration rate for a medium-sized barchan dune, in the northern Rigboland sand sea, of approximately 6.5 m/yr. However, he emphasized that any changes in size and topographic position can directly affect barchan sand dune migration rate. Similar investigations on the direction and magnitude of sand dunes (in particular barchan dunes) and overall sand sea movements have been undertaken by other researchers^13, 21–25^. Their findings show that movements vary according to climatic parameters (wind speed and direction), topographic position, sand availability, the type and amount of vegetation cover, as well as desertification processes affecting the margins of arid lands and deserts. Although the direction and rate of sand transport are mostly governed by the wind regime, direction and speed, the research reported here shows that the location of available sand sources is also important.

**Figure 6:**
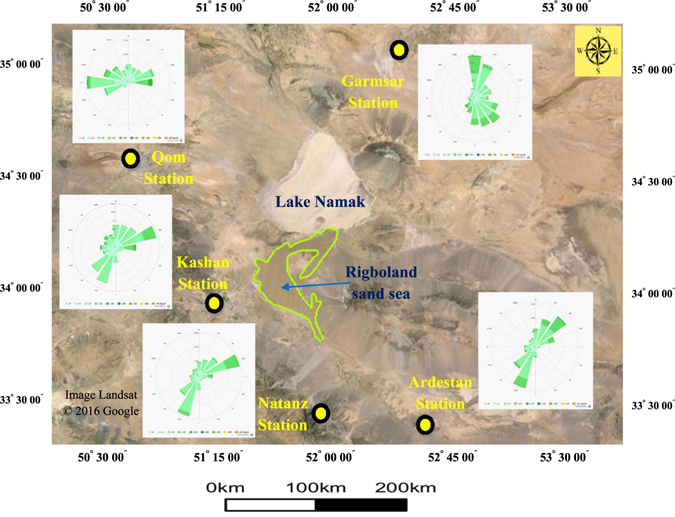
The location of five weather stations around the Rigboland sand sea. The wind roses show the annual distribution and magnitude of wind direction and speed (Image from Google Earth Software).

The nearest synoptic meteorological station to the Rigboland sand sea is in Kashan city, to the west. Average annual precipitation is approximately 130 mm and most rainfall is in March and April. Wind direction and wind speed are key parameters that control the aeolian processes. At the Kashan weather station, wind directions cluster in the ENE (56–79°) and SSW (191–214°), with maximum wind speeds ranging from 5–19 km.h^−1^. Wind roses for regional cities around the Rigboland sand sea are shown in Fig. 6.

As seen in Fig. 6, the most erosive (highest speed) winds for the western margin of the Rigboland sand sea at Kashan, Natanz and Qom stations are from eastern and north-eastern directions, with lower velocity winds from the west or southwest. Contrary to this, the most erosive winds to the east of the Rigboland sand sea are mostly from the north at Gamsar and from the south-west at Ardestan, again with lower velocity winds generally in the opposing directions. Due to these multi- and bimodal wind regimes around the Rigboland sand sea, the rate of sand sheet formation is high and the contained dunes are commonly longitudinal. The multimodal wind regime also results in lower dune height and greater dune mobility. These wind parameters may help explain the observed expansion and contraction in area and volume of the Rigboland sand sea.

**Figure 7:**
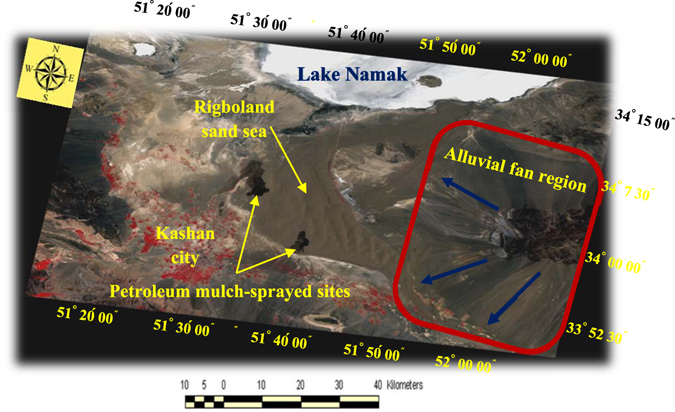
Landsat_TM_ image showing alluvial fan regions (red rectangle) and direction of sand supply (blue arrow) supplying sand to the expanding eastern margins of the Rigboland sand sea. Also shown are petroleum mulch-sprayed sites marginal to Kashan city, designed to prevent sand dunes from encroaching (ENVI software version 5.1).

Supply of sand is also an important factor in the overall development of the sand sea. The most available external sand sources for the Rigboland sand sea are to the northeast and southeast (Fig. 5, yellow-colored parts), originating in alluvial fans, which contain large boulders, gravel, sand, silt and clay. In these fans, there is a general decrease in particle size downstream with abundant sand-sized particles of physically weathered granite, rhyolite, dacite, red conglomerate, sandstone and other silicate rocks, derived from up-catchment exposures. Glennie^26^ has noted that in many desert areas, an important source of sand particles is in the sediments deposited in alluvial fans shedding off nearby mountainous highlands. Typically, around 60 percent of this type of sediment consists of sand-sized particles^27^. This is consistent with the observation from this study that geomorphological units such as piedmonts, pediments and alluvial fan facies marginal to eroding mountains in the eastern upstream areas of the Rigboland sand sea are playing a key role in its expansion along the northeastern and southeastern margins (Fig. 7). Satellite imagery of landforms on the eastern margin of the Rigboland sand sea also reveals significant areas of disrupted drainage related to sand addition and dune encroachment. The extent of this disruption suggests sand movement to this area from the east over a considerable period of time.

As Fig. 5 shows (green areas), the area of the Rigboland sand sea has been reduced in some of the eastern parts where there are sediments derived from alluvium and alluvial fan deposits. Field observations clearly show that this area contains sandy textured soils with a high moisture content and hence good vegetation cover. This has resulted in a significant decrease in sand dune encroachment. In the western area of the Rigboland sand sea, near Kashan city, there are currently stable lands that do not display tangible encroachment due to some biological and mechanical initiatives to combat desertification. Additional to these remediation methods, some land-use changes on the sand sheets and sandy plains, such as urbanization, construction of industrial zones and other development projects have reduced the apparent areas of encroachment. The spraying of petroleum mulch on shifting sand dunes has had some impressive results in effectively fixing and stabilizing the sand dunes, as clearly seen in Fig. 7. Most of the remediation efforts are directed towards protecting Kashan city, a major and vital city in central Iran with a population of 300,000.

Generally, the natural and anthropogenic apportionment of the sand dune encroachment and desertification processes in the Rigboland sand sea has not been clearly quantified, although similar research in other regions of the world by refs 28–31 have considered this issue. Qualitative observations on a comprehensive scale from the current study show heterogeneous and asymmetric changes that can be related to anthropogenic effects, particularly around settled areas. These changes have involved both expansion and shrinkage of marginal areas of the Rigboland sand sea. The key drivers for these anthropogenic changes include over-grazing of naturally poor rangelands with low vegetation cover, changes in land use and land cover and remediation efforts. It is also possible that intensive groundwater withdrawal leading to water table lowering and drying of the regolith could assist desertification. These anthropogenic changes overly and may be excacerbated by natural factors of wind erosion, sheet flow from periodic torrential rains and availability of sand supply. Abandoned villages and agricultural lands present in the study area, particularly in the southeastern parts, give a good indication of the serious threat of sand dune movement and desertification processes.

Some anthropogenic activities have significantly reduced sand movement and desertification locally, as well as providing societal benefits. These activities include urban and industrial development, government infrastructure construction, irrigated agriculture and surrounding, protective remediation. The effects are most noticeable on the sand sheets and sandy plains of the western parts of the Rigboland sand sea. Associated societal benefits include a decrease in rural to urban migration and higher income levels. It is generally accepted that where both natural and anthropogenic processes contribute to environmental hazards, controlling the anthropogenic activities is more feasible. Therefore, it is strongly advised that human activities around the sand sea be monitored and carefully managed to help prevent its expansion.

This study shows that regional-scale, natural changes to the Rigboland sand sea, both expansion and contraction, are largely the result of wind regimes, sand supply and its transport, particularly from suitable geomorphologic and lithological source areas. It is important to recognize that hydro-aeolian processes have been important in the source areas for the sand (cf. ref. 32), whose research indicated sediment from fluvial and alluvial sources reach the dune fields in the northeastern sector of Owens Lake in California.

Unfortunately, little is known about how active or non-active sand dunes happen to be across Iran, and complementary studies on the age and origin of the Iranian sand seas have not yet been precisely carried out. So geomorphic, luminescence dating and chrono-stratigraphic data are vital in future studies. One study has indicated that the playa sediments from the Iranian Plateau show evidence for increased concentrations of aeolian sands between 22 and 12 ka^33^.

Combining RS analysis with field observations and measurements is vital for a better quantitative understanding of desertification processes and sand dune encroachment as emphasized by refs 34–37. The current study provides useful knowledge on the Rigboland sand sea in central Iran, particularly in regard to which areas have potential for shifting sand dunes. The results provide more accurate information for risk assessment of sand dune and sand sea movement and encroachment.

## Conclusion

- Time series remote sensing (RS) analysis using a cross tabulation technique, combined with field-based surveying has demonstrated expansion and shrinkage of the Rigboland sand sea in different directions and with variable magnitude rates.
- The Eastern Rigboland sand sea is fed by sand-sized particles derived from alluvial fan systems on the margins of an eroding upland with bedrock types including granite, limestone, basalt and andesite.
- Both biological and mechanical initiatives to combat desertification by stabilizing sand dunes in the Rigboland sand sea have been effective at preventing dune movement and encroachment.

## Methods

### Data set

This study examines a ten-year time span (from 1988 to 1998) utilizing two Landsat_TM_ images to calculate the changes in the Rigboland sand sea. The multispectral scanning radiometer of the Landsat Thematic Mapper (TM) is an advanced, multispectral Earth resources sensor designed to achieve higher image resolution, sharper spectral separation, improved geometric fidelity and greater radiometric accuracy and resolution than the MSS sensor.

### Satellite imagery pre-processing

A set of corrections were applied during Landsat_TM_ image pre-processing. These included: corrections related to compensating for the distortion created by off-axis projector or screen placement or non-flat screen surface (geometry correction); sensitivity of the remote sensor; topography and sun angle; and atmospheric scattering and absorption (radiometric calibration). Correction also included removing the effects of the atmosphere on the reflectance values of ground images (atmospheric correction).

Because of the key role of the geometry correction for change detection, this correction was made on the basis of a pixel-by-pixel, image to image technique. For increasing the accuracy of geometry correction, ground control points (GCP_s_) were utilized. Atmospheric correction through Dark Pixel Subtraction (DPS) was applied and assumed that the darkest objects in the images should have a Digital Number (DN) of zero. Minimum pixel values from each band were established, using histograms and this value subtracted from all of the pixels in the bands.

### Spectral bands used and image classification technique

Although the spatial resolution of spectral bands is of key importance in selecting the most useful spectral bands, less appreciated is how changes in irradiative energy reflected by different surface materials can be used to identify features of interest. To tackle this issue, a correlation table of Landsat_TM_ spectral bands was applied and spectral correlation between each band used to show the varying capability of various spectral bands to indicate features. It should be noted the lowest correlation between spectral bands has the highest feature diagnosis.

To classify the features in the Landsat_TM_ images, the process of image enhancement for visual interpretation was performed to identify homogeneous groups of pixels, which represent various features or land cover classes of interest in the Rigboland sand sea satellite images. The classification procedure applied is a supervised method. The selection of appropriate training areas was conducted based on the analyst’s familiarity with the geographical area and the knowledge of the actual surface cover types present in the image. Field work was also used to record more than 250 ground control points (GCP_s_) using GPS across the region. These points were used to check on the accuracy and precision of classification in the imagery. The most powerful classifier in common use, namely Maximum Likelihood, was applied. Both the variances and covariances of the class signatures were employed when assigning each cell to one of the classes represented in the signature file and bell-shaped Landsat_TM_ spectral bands of the region.

### Change detection technique

After all the pre-processing operations, a change detection technique using a Cross-Tabulation module was employed. Each pixel of the first image with given class was compared with each pixel of the second image, to determine the amount of change over all classes. Differences between two images with two different acquisition dates could thus be highlighted. This means that some landforms in image 1 (e.g. agricultural land) have changed to other landforms (e.g. sand dune) in image 2 and vice versa. This method gives a good indication of the chance of change in an area and also an approximate idea of the direction. Therefore, this is a rather useful tool.

The KAPPA Index of Agreement (KIA) is a means to test two images to determine if their differences are due to ‘chance’ or a ‘real (dis)agreement’. It is commonly used to check for accuracy of classified satellite images versus some ‘real’ ground-truth data^38, 39^ (Formulas 1 and 2).

1 $${\boldsymbol{K}}=\frac{{Obsrved}\,{Accuracy}-{Chance}\,{Agreement}}{1-{Chance}\,{Agreement}}$$

2 $${\boldsymbol{K}}=\frac{{N}{\sum }_{{i}=1}^{{r}}{{x}}_{{ii}}-{\sum }_{{i}=1}^{{r}}({{x}}_{{i}+}\ast .\,{{x}}_{+{i}})}{{{N}}^{2}-{\sum }_{{i}=1}^{{r}}({{x}}_{{i}+}\ast .\,{{x}}_{+{i}})}$$

Where:

K: Overall Kappa coefficient

r: Number of row in cross classification table

x_ii_: Number of combinations along the diagonal

x_i+_: Total observations in row i

x_+i_: Total observations in column i

N: Total number of cells

In this research, ENVI 5.1, IDRISI Kilimanjaro and Arc GIS 10.2 were used to calculate all of those processes and operations.
